# Intratumor Heterogeneity in Hepatocellular Carcinoma: Challenges and Opportunities

**DOI:** 10.3390/cancers13215524

**Published:** 2021-11-03

**Authors:** Sharanya Maanasi Kalasekar, Chad H. VanSant-Webb, Kimberley J. Evason

**Affiliations:** Department of Pathology and Huntsman Cancer Institute, University of Utah, Salt Lake City, UT 84112, USA; sharanya.kalasekar@hci.utah.edu (S.M.K.); Chad.vansant-webb@hci.utah.edu (C.H.V.-W.)

**Keywords:** cancer, liver cancer, single-cell sequencing, β-catenin, TERT, intratumor heterogeneity, therapy resistance

## Abstract

**Simple Summary:**

Hepatocellular carcinoma (HCC) is a deadly form of liver cancer that has poor patient outcomes and survival. Unlike many other cancers, HCC has not seen the benefit of individualized treatment. Part of the reason HCC is hard to treat is due to differences among distinct regions of each tumor, also known as intratumor heterogeneity (ITH). In this review, we summarize what is known about ITH in HCC, describe how it influences tumor behavior and treatment strategies, and discuss future research directions for ITH and HCC.

**Abstract:**

Hepatocellular carcinoma (HCC) represents a leading cause of cancer-related death, but it remains difficult to treat. Intratumor genetic and phenotypic heterogeneity are inherent properties of breast, skin, lung, prostate, and brain tumors, and intratumor heterogeneity (ITH) helps define prognosis and therapeutic response in these cancers. Several recent studies estimate that ITH is inherent to HCC and attribute the clinical intractability of HCC to this heterogeneity. In this review, we examine the evidence for genomic, phenotypic, and tumor microenvironment ITH in HCC, with a focus on two of the top molecular drivers of HCC: β-catenin (CTNNB1) and Telomerase reverse transcriptase (TERT). We discuss the influence of ITH on HCC diagnosis, prognosis, and therapy, while highlighting the gaps in knowledge and possible future directions.

## 1. Introduction

Hepatocellular carcinoma (HCC) is a leading cause of cancer-related mortality [1]. Regardless of etiology, HCC has few druggable targets, which makes treatment of advanced HCC challenging. Patients with early-stage HCC benefit from surgical interventions which can be curative, whereas late-stage patients are treated with locoregional treatments like chemoembolization and/or systemic chemotherapeutics. The multikinase inhibitor sorafenib extends survival by 4.3 months (95% CI, 4.0 to 5.6), and the combination of the vascular endothelial growth factor (VEGF) inhibitor bevacizumab and the immune checkpoint inhibitor atezolizumab extends overall survival by 6.8 months (95% CI, 5.7 to 8.3) [2]. Not only do these first-line chemotherapies only modestly improve survival, but molecular testing for guiding HCC treatment is used little, if at all [3], underscoring the need for new drug development strategies in HCC.

Intratumor heterogeneity (ITH) influences tumor progression, aggression, therapeutic resistance, and disease relapse across cancer types [4,5]. Although cancer was considered a clonal disease for several decades, ideas of genomic diversity within single tumors emerged in the 1950s [4]. Subsequently, mouse mammary tumor studies in the 1970s and 1980s documented phenotypic heterogeneity, with cells from different sections of the same tumor varying in growth rate, immunogenicity, ability to metastasize, and response to drugs [6]. The first studies demonstrating clonal interaction and cooperation emerged in the 1980s and 1990s [6]. The widespread use of next-generation sequencing (NGS) and other computational tools on multiregional and liquid biopsies have propelled in-depth investigation into the tumor type-specific and context-driven characteristics of ITH.

In this review, we examine the landscape of evidence for genomic and phenotypic ITH in HCC and explain how this ITH might influence tumor behavior, including via clonal evolution. We next focus on describing the role of ITH in two of the top molecular drivers of HCC: β-catenin and TERT. Finally, we discuss the influence of ITH on HCC diagnosis, prognosis, and therapy.

## 2. How Tumors Become Heterogeneous

**Figure 1:**
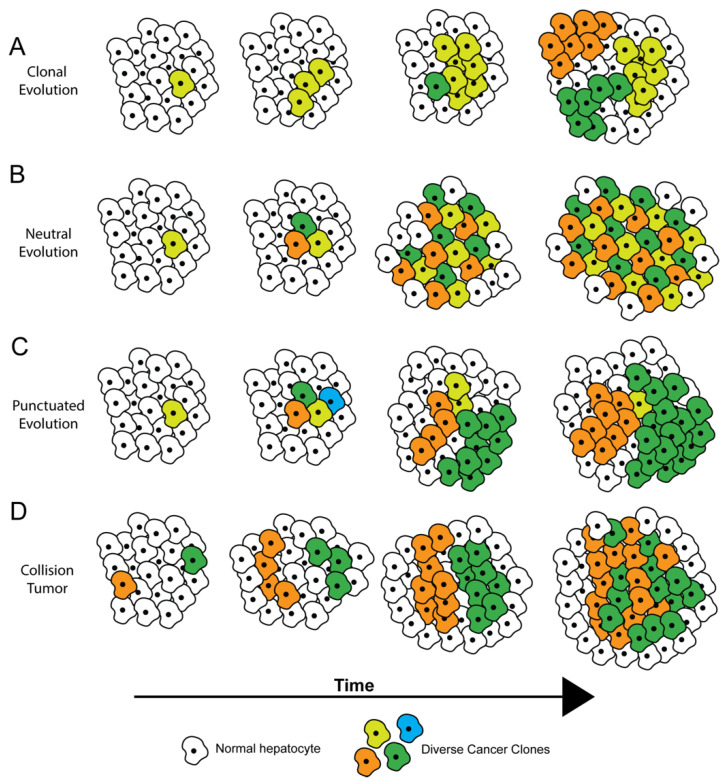
Theories of tumor evolution. (**A**) Clonal evolution, where all tumor cells diverge from a common ancestor with a fitness advantage, and subclones emerge with acquired genomic alterations. (**B**) Neutral evolution, where mutations randomly accumulate over time with no one population having a selective advantage. (**C**) Punctuated evolution, where a large number of genetic alterations occur at a point in time, but only a few dominant clones persist. (**D**) Collision tumor, where physically distant clonal cell populations converge as they proliferate, eventually colliding and forming a single tumor.

Several theories have been proposed to describe how ITH arises, most of which are based on tumor evolution [7,8] (Figure 1). Within tumors, cells genetically identical to each other are considered clones. Subclones arise from clones when new genomic aberrations lead to distinct subpopulations of cells. Clones and subclones are identified by their driver and passenger mutational profiles. Driver mutations in critical oncogenic or tumor-suppressor genes confer a fitness advantage to cells. In theory, if a driver mutation is present during the first tumor-initiating event, then this driver mutation will be present in all cells and subclones within the tumor, with a cancer cell fraction (CCF) of 1. Such mutations are called trunk mutations as they precede any branching nodes on a phylogenetic tree representing the evolution of the tumor. During subsequent tumor progression and evolution, additional driver mutations may arise, which further increase the tumor’s fitness; passenger mutations generally do not affect tumor fitness. In either instance, cellular alterations with CCF less than 1 are considered subclonal [9].

Definitions of clones and subclones apply more to single biopsies than to multiple biopsies taken over time. Clonal mutations in a biopsy may become subclonal or even absent in subsequent biopsies. This phenomenon can result from sampling bias or because of an evolving tumor with increasing genomic ITH [9].

Within a tumor and across time, subclonal diversification and tumor evolution have been proposed to occur via various possible trajectories (Figure 1) [7], which have implications for diagnosis and treatment. A tumor can undergo diversification through clonal evolution (Figure 1A), where all tumor cells diverge from a common ancestor with a fitness advantage, and subclones later emerge. In this case, therapies targeting mutations in the common ancestor (trunk mutations) can prove the most beneficial, and a single biopsy may suffice to identify the trunk mutation(s). In neutral evolution (Figure 1B), mutations randomly accumulate over time with no single clonal or subclonal population having a selective advantage. Tumors with neutral evolution may prove hard to treat with monotherapies because at least one of these subclonal populations is likely to survive any single treatment. During punctuated evolution (Figure 1C), a large number of genetic alterations occur at a point in time, but only a few dominant clones persist because of inherent or exogenously driven (drug treatment) selection pressure. These tumors may be effectively targeted by treatment regimens aimed towards the dominant clones. Robust lineage-tracing and tumor evolution-calculating computational algorithms have recently been developed to help determine which evolutionary trajectories are most applicable to a given tumor [10,11,12]. Additional clinical studies will be helpful to see if these algorithms and accompanying statistical tools are able to improve therapy design for individual cancer patients, including those with HCC.

In addition to subclonal diversification, another potential source of ITH is collision between two or more independently arising tumors (Figure 1D). Collision tumors may be more common in the liver than in other organs because the vast majority of HCCs arise in the setting of chronic hepatitis, wherein the entire liver is subject to a cancer-promoting field effect driven by the inflammatory and regenerative milieu. Xu et al. performed hierarchical clustering of whole exome sequencing (WES) and transcriptomics-generated mutational signatures from six patients with multifocal HCC, with each patient showing some degree of ITH [13]. In one patient they showed that the sample from a single region of the tumor possessed a remarkably distinct mutational signature compared to the other regions, suggesting that this sample may be representative of a tumor of independent origin (Figure 1D).

## 3. Subtypes of Genomic ITH

Genomic ITH may arise from a plethora of different types of alterations to the cells’ genetic material, including somatic mutations, epigenetic changes, and large-scale genomic alterations.

Somatic genomic mutations are perhaps the most well-studied source of genomic ITH; the extent of this type of genomic ITH is variable among tumors but tends to increase in more advanced HCC. Sirivatanauksorn et al. used arbitrarily primed PCR amplification-based DNA fingerprinting of two or more regions of 31 HCCs and found that the genomic fingerprint was homogeneous in HCCs less than 6 mm, indicating minimal ITH. However, HCCs larger than 6 mm showed distinct genomic fingerprints, even when derived from synchronous HCCs, suggesting increased ITH and clonal evolution in more progressed HCCs [14]. Performing NGS analysis of multiple liver lesions (primary and intrahepatic metastases) and tumor thrombi from 10 patients, Xue et al. showed that the percentage of mutations shared by all tumors within each patient ranged from 8% to 97% [15]. These findings indicate that the extent of genomic ITH within anatomically and temporally distinct HCC regions varies widely among patients [15]. In line with Sirivatanauksorn et al., HCCs greater than 5 cm displayed significantly higher genomic ITH than smaller HCCs, providing further evidence for the increase in genomic ITH with tumor progression.

The location of somatic mutations on the phylogenetic tree of tumor evolution has implications for diagnosis and treatment response. Coding mutations in *Tumor protein 53 (TP53)* and *β-catenin (CTNNB1)*, along with promoter mutations in *Telomerase reverse transcriptase (TERT)* that increase *TERT* transcript levels, were the most frequent trunk mutations in 151 samples of HCC in various stages of tumor progression [16]. A total of 51% of dysplastic nodules and small HCCs had a mutation in at least one of these genes, and 80% of these mutations were present in both primary and metastatic tumors in advanced HCC [16]. These findings suggest that these mutations are early and possibly tumor-initiating events, and diagnostic information on major drivers and trunk mutations could be captured via single-region biopsies in many cases. However, in some patients, aberrations affecting treatment efficacy may be present only in tumor subclones. Xu et al. identified one patient with three sorafenib-targeted branch alterations, predicted to decrease drug response, that were present in subclones but not in the trunk [13]. Overall, Xu et al. found that five out of six patients with multifocal HCC had sorafenib-resistant subclones in one or more foci, which would theoretically render treatment with sorafenib ineffective in these individuals.

**Figure 2:**
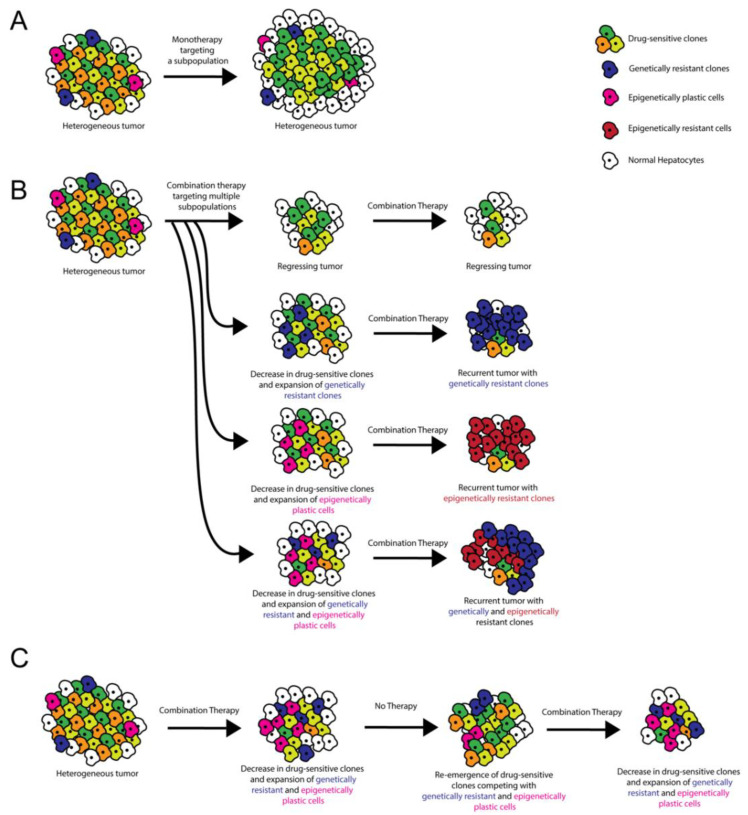
Role of ITH in targeted therapy resistance and treatment stratagem. (**A**) Monotherapies target a subset of tumor cells, resulting in incomplete tumor attrition. (**B**) Combination therapies target multiple oncogenic pathways, potentially resulting in tumor regression. However, recurrent tumors can occur when pre-existing ITH or ITH induced by cell–cell/cell–microenvironment interactions results in the emergence of genetically and/or epigenetically resistant clones. (**C**) Combination therapy administered with alternating periods without treatment allows for the disappearance and re-emergence of drug-sensitive clones. Competition between clones limits the emergence of resistant clones.

There is substantial evidence from non-liver cancers to show that certain driver mutations display a tendency to be subclonal in nature. Mutations in *PIK3CA*, for example, have been found to be more prevalent in subclonal populations in colorectal carcinoma [17], non-small-cell lung carcinoma [18], melanoma [19], clear cell renal carcinoma [20], breast [21], and ovarian cancers [22]. In HCC *PIK3CA* is mutated in 28% [23] to 35.6% [24] of cases, but there are limited studies defining the percentage of cells with *PIK3CA* mutations within each tumor. In a patient with five HCC tumors, Xu et al. identified a subclonal mutation in *PIK3CA* (p.E545K) in only the two smallest tumors [13]. Janku et al. found that two patients with putatively activating mutations in the PI3K/AKT/mTOR pathway (*PIK3CA* and *NF1*) had therapeutic benefits from targeted combination therapy with mTOR inhibitors (RECIST −30% and −15%, respectively) [25]. The percentage of tumor cells with *PIK3CA* mutations was not defined in these cases, and it is not clear how widespread mutations in *PIK3CA* must be to influence therapeutic response in HCC. Nonetheless, given the tendency for *PIK3CA* mutations to be subclonal, these studies highlight the potential benefits of taking multi-regional biopsies to help inform clinical decisions (Figure 2). Utilizing only a single-region biopsy has the potential to mis- or underutilize targeted therapies [13,25].

In addition to somatic mutations, epigenetic processes such as DNA methylation, histone modification, and chromatin remodeling can also contribute to genomic ITH [26,27,28]. Methylation profiling of multiple samples from five patients with HCC revealed ITH with respect to CpG methylation, which ranged from 2% to 64% [29]. Hypermethylated regions were enriched for cancer-related genes involved in proliferation, differentiation, death, migration, adhesion, and transcriptional regulation [29]. Lin et al. used a mathematical algorithm called Mutant-Allele Tumor Heterogeneity (MATH) [30,31] to estimate the genetic ITH from bulk tumor whole exome sequencing data from 377 HCCs in The Cancer Genome Atlas (TCGA) [29]. Although the TCGA cohort contains data from only single-region samples, they used MATH to predict a methylation ITH (mITH) score for each patient in the cohort. Patients with high mITH scores displayed a tendency, albeit statistically insignificant, to have worse survival outcomes than those with lower mITH scores. This disparity attained significance when comparing the survival outcomes of patients with the highest (top 10%) and lowest (bottom 10%) mITH scores. The mechanism by which high mITH promotes hepatocarcinogenesis is not clear, but it could involve clonal cooperation between differentially methylated subsets of tumor cells (see below).

Large-scale genomic alterations are another important source of genomic ITH. Copy number heterogeneity is highly prevalent in renal clear cell carcinoma and contributes to mutational ITH in high-grade serous ovarian cancer and esophageal adenocarcinoma [4]. While chromosomal instability and the resultant transcriptional alterations are prevalent in HCC and have been correlated to worse prognosis [32], the extent of copy number ITH in HCC has not been well-characterized.

## 4. Phenotypic/Functional ITH

Phenotypic or functional ITH in cells refers to differences in behavior among cells within a tumor. It can result from differential expression of a heterogeneous genome, heterogeneous post-transcriptional/translational modifying mechanisms across tumor cells, or varying levels of exposure to environmental factors such as hypoxia, stress, and therapeutic agents. An example of functional ITH is when genetically identical tumor cells respond differentially to epithelial–mesenchymal transition (EMT)-inducing signals like TGF-β and switch between epithelial, mesenchymal, or hybrid E/M states [33]. Such plasticity allows tumor cells to adapt quickly to microenvironmental stressors and to progress through selection pressures [33].

Studies by Ho et al. and Zheng et al. have elegantly demonstrated the presence of phenotypic ITH in HCC. Using single-cell sequencing, Ho et al. showed that single cells isolated from patient-derived tumor xenografts from human HCC could be divided into two subpopulations with similar mutational signatures based on epithelial cell adhesion molecule expression (EpCAM+ or EpCAM−), indicating that these two populations differed at the level of the transcriptome [34]. Genes involved in lipid metabolism were upregulated in EpCAM+ cells, whereas genes involved in translation and RNA processing were upregulated in EpCAM− cells. The EpCAM+ cells also encompassed a rare population of CD24+/CD44+ cells, which were enriched in stemness-related gene expression. Although the two populations of cells had similar mutational signatures, there was observable ITH with regard to the transcriptome, which had a direct impact on cellular behavior and viability.

Zheng H. et al. characterized cancer stem cell (CSC) heterogeneity in HCC. They showed that Huh1 and Huh7 cell lines demonstrated varying CSC marker expression (CD133, CD24, EpCAM) when cultured in monolayers or three-dimensional organoids [35]. They also found that only 15% to 20% (Huh1) or 8% to 12% (Huh7) of single-marker-positive cells had self-renewal capability, suggesting ITH with respect to self-renewal capacity in cells expressing the same marker. Zheng et al. further found that cells arising from single-marker positive populations or triple-negative CSCs, when cultured, could each give rise to a population of mixed CSCs. These data suggest the generation of heterogeneity within relatively homogeneous populations (single-marker-expressing or triple-negative CSCs), indicating phenotypic plasticity. Thus both Ho et al. and Zheng et al. showed phenotypic ITH with respect to stemness in HCC, but the importance of this type of ITH to HCC genesis, maintenance, and behavior is not entirely clear.

Studies in breast and lung cancer indicate that expression of microRNAs (miRNAs) and other noncoding RNAs (ncRNAs) can be heterogeneous within tumors. In breast cancer, Veryaskina et al. compared the expression of miRNAs of multiple regions of breast cancer and found varying levels of ITH among 10 of the 16 miRNAs analyzed [36]. Similarly, Stewart et al. utilized circulating tumor-cell-derived xenografts and single-cell RNA sequencing to investigate ITH in small-cell lung cancer (SCLC) [37]. They found significant ITH with respect to gene expression profiles, including variable expression of ncRNAs such as *MALAT1* [37]. This finding in SCLC may also be relevant to liver cancer because upregulation of *MALAT1* has been associated with poor prognosis in HCC [38], although the extent of *MALAT1* ITH in HCC is not known. Together, these publications indicate significant ITH of ncRNA expression in diverse tumor types. Future clinical studies will be required to determine the mechanism by which ITH of ncRNA expression arises and how it influences HCC behavior.

## 5. Tumor Microenvironment ITH

Variations in stromal cells represent another important type of ITH. HCC occurs and progresses in the context of a dynamically evolving tumor microenvironment that includes tumor-resident stem/progenitor cells, activated hepatic stellate cells, carcinoma-associated fibroblasts, myofibroblasts, endothelial cells, pericytes, dendritic cells, and tumor-infiltrating immune cells [39]. The development of innovative, more effective therapies for HCC requires an improved understanding of the entire hepatic milieu, which forms the objective of some recent investigations.

Most studies examining tumor microenvironment ITH have focused on immune cells. Through single-cell transcriptomic profiling of 5063 T-cells from peripheral blood, tumor, and adjacent normal tissues of six patients with HCC, Zheng et al. identified 11 distinct subtypes of tumor-infiltrating lymphocytes (TILs) [40]. Tregs and exhausted CD8+ T-cells accumulated within HCC tumors; 82% of the Treg cells were unique and did not share T-cell receptors (TCRs) with tumor CD4+ cells or adjacent normal tissue Tregs, thus demonstrating T-cell ITH [40]. Kurebayashi et al. characterized the tumor immune microenvironment of 919 regions of 158 HCCs and found that the tumor microenvironment could be classified based on B- and T-cell infiltration levels into three immune subtypes—high, medium, and low. Although all patients could be categorized into one of the three immune subtypes based on the predominant pattern, over 50% of patients exhibited ITH with respect to these subtypes [41].

Losic et al. leveraged a combination of RNA sequencing, DNA sequencing, T-cell receptor sequencing, and single-nucleotide polymorphism array data to characterize cancer-cell-immune cell interactions across multiple regions of HCC specimens from 14 patients [42]. In three patient tumors, the authors observed significant ITH in tumor infiltrating lymphocyte (TIL) burden in different regions of the tumor, as measured by the number of RNA-seq reads that mapped to the VDJ loci. Tumor regions from two patients also showed ITH with respect to TIL architecture and the presence of tertiary lymphoid structures. These structures are correlated with a lower risk of HCC recurrence [43], thereby implicating a prognostic value for immune cell ITH. The authors next investigated the relationship between tumor cell mutational status and immunogenicity by using in silico predicted neoepitope–HLA allele binding affinities to quantify the likelihood of neoepitope presentation and recognition by a T-cell. In five out of 11 patients, these binding affinities varied significantly between regions from the same tumor, indicating that tumor cell mutational heterogeneity likely underlies heterogeneity in T-cell response. In one patient, branch mutations were associated with significantly higher immunogenicity scores compared to trunk mutations, in line with the notion that early driver mutations are immune-evasive.

Tumor microenvironment ITH can potentially influence treatment eligibility in cancer patients. In a recent study, Shen et al. used subdivided single-sample biopsies to analyze ITH within the immune tumor microenvironment [44]. They performed IHC and RNAseq on 77 regional samples from 13 individual HCC biopsies, 12 of which were of viral etiology. One of the eight IHC markers used was programmed death ligand 1 (PD-L1), a marker expressed by cancer cells to inhibit the immune system. Their study identified eight cases (62%) which had no PD-L1 expression in any tumor sample, one case (7%) which had homogenous PD-L1 expression in all regional samples, and four cases (30%) which had heterogeneous PD-L1 staining between tumor regions. Furthermore, they noted regional differences in tertiary lymphoid structures in 57% of cases [44]. Overall, the study found a single biopsy could accurately survey the tumor immune microenvironment in 60% to 70% of cases of HCC. Biopsy of cases with heterogeneous PD-L1 staining could potentially miss PD-L1 expression, affecting treatment decisions. While PD-L1 expression is not yet used in HCC to determine eligibility for immunotherapies, Food and Drug Administration (FDA) approvals for immune checkpoint inhibitor therapy are linked to specific levels of PD-L1 expression for several other cancers including non-small-cell lung cancer and bladder cancer [45].

HCC is a highly vascularized malignancy [46], but there are conflicting data with regard to microvessel density (MVD) and microvascular invasion (MVI) as predictive indicators for patient prognosis [46,47]. One possible explanation for these discrepant results is that the amount of vascular ITH may influence tumor behavior independent from the degree of MVD or MVI. Two recent imaging studies have addressed this possibility, using coded harmonic angio ultrasound (CHA) [48] and fractal density analysis of contrast enhanced-CT (CE-CT) [49] to analyze the inter- and intratumoral vasculature. Utilizing CHA to compare liver nodules of HCCs, hepatic metastases, and benign lesions, Jang et al. identified that the majority of HCCs (69%) had a heterogeneous, “irregular branching” pattern, and 27% showed “random stippling” [48]. The “irregular branching” pattern was seen exclusively in HCCs, and CHA could clearly distinguish HCCs from other hepatic nodules. In a phase II clinical trial, Hayano et al. used CE-CT with fractal density analysis to identify ITH and to determine the effects of sunitinib on intratumoral vasculature [49]. They found that patients with favorable progression-free survival had significantly lower fractal density (less ITH and less abnormal vasculature) at baseline. Furthermore, Hayano et al. determined that greater reduction in fractal density post-treatment with sunitinib significantly correlated with better progression-free survival and overall survival. Together, these studies support the hypothesis that vascular ITH is present in HCC and influences tumor behavior.

Cancer-associated fibroblasts (CAFs) shape the extracellular matrix and are key contributors to ITH. CAFs display an array of different phenotypes, cell surface markers, and functions reflecting diverse potential origins including activated hepatic stellate cells [50], mesenchymal stem cells [51], and endothelial cells [52]. CAFs are usually considered tumor-promoting, but they can be tumor-suppressive as well [53], indicating marked heterogeneity in function.

The effect of CAFs on tumor behavior may be dependent on which cell surface marker(s) they express. While no cell surface marker is specific to CAFs, they typically display some combination of α-smooth muscle actin (α-SMA), fibroblast activating protein (FAP), and/or vimentin [54]. When staining CAFs for α-SMA, Takamura et al. found an inverse correlation between α-SMA staining and both disease-free survival and overall survival in HCC patients [55]. A similar study with a larger cohort also found an inverse correlation between α-SMA staining and disease-free survival [56]. However, Kim et al. did not find an association between FAP-positive CAFs and HCC prognosis [57]. It is possible that different patient characteristics or methodologies could account for the contradictory results among these three studies. However, these reports also raise the intriguing possibility that α-SMA-expressing CAFs could have stronger tumor-promoting effects than FAP-expressing CAFs, supporting heterogeneity in CAF function. This hypothesis could be tested by examining the correlation between prognosis and different CAF markers including α-SMA and FAP in the same set of HCC patients.

## 6. Clonal Cooperation

Genomic, phenotypic, and ITH heterogeneity may impact tumor behavior via clonal cooperation, in which distinct groups of tumor cells show enhanced aggressiveness when growing together than when existing apart. Clonal cooperation is essential for tumor maintenance and progression in diverse cancer types including brain and breast cancer [6]. For example, co-culturing of genotypically and phenotypically distinct subclones from the same gliomas resulted in enhanced invasion and migration in the subclone with poor motility [58]. In vivo, these differentially labeled subclones retained their mixed proportions and infiltrated the central nervous system more extensively than one subclone alone, suggesting a tumor-enhancing cooperation between these subclones.

Paracrine or juxtracrine excretion and signaling of growth factors between phenotypically diverse groups of cancer cells can influence tumor behavior to create and maintain ITH within tumors. In mouse mammary tumors consisting of a Wnt1-producing, Hras wild-type luminal subclone and an Hras-mutant basal subclone, it was found that both subclones were necessary for tumor propagation, with a critical dependence on luminally produced Wnt1 [59]. In another recent study, Li and Thirumalai showed that unequal distribution of paracrine factors between cells that make them and cells that consume them may be required for the emergence of stable ITH [60]. In “harsh” conditions with a lack of exogenous insulin-like growth factor II (IGF2), co-operation between cell populations was observed, and thus ITH was maintained. However, when cells were provided a surplus of exogenous IGF2, co-operation was lost, and competition was observed between cell populations. From these findings, Li et al. were able to generate predictive models of ITH in glioblastoma multiforme [60].

The above studies demonstrate that phenotypic ITH can promote tumor aggressiveness via clonal cooperation in brain and breast cancer. Assays combining phenotypically distinct groups of HCC cells in varying ratios and implanting them in animal models could be helpful to define the importance of clonal cooperation to HCC behavior. The platforms developed by Ho et al. [34] and Zheng et al. [35] could be useful in this regard.

## 7. ITH of TERT and Wnt/β-Catenin Signaling

Genomic and phenotypic heterogeneity of specific oncogenes is an important source of ITH in HCC. The most commonly mutated genes in HCC include *TP53* (~30% of cases) [61], *CTNNB1* (11% to 37% of cases) [62], and *TERT* (~60% of cases) [61]. Evidence to date indicates genomic *TP53* ITH is not prevalent in HCC. In a meta-analysis by Huang et al. of five studies, encompassing 36 patients with HCC, 72.2% of cases harbored a somatic mutation in *TP53*, all of which were identified as trunk mutations [63]. These data indicate that *TP53* mutations are generally not branch mutations, and thus have less potential for genomic ITH. ITH of *TP53* expression has been noted in a variety of tumor types including melanoma and colon cancer [64], but phenotypic ITH of *TP53* has not been well characterized in HCC. Thus, our discussion of genomic and phenotypic ITH of specific oncogenes in HCC focuses on two other major players, *CTNNB1* and *TERT.*

### 7.1. Wnt/β-Catenin Pathway ITH

The Wnt/β-catenin signaling pathway is a critical regulator of organismal development and oncogenic transformation [65,66,67]. In the presence of Wnt ligands, the adenomatous polyposis coli (APC)/AXIN/glycogen synthase kinase-3β (GSK-3β) degradation complex is inhibited, stabilizing cytoplasmic β-catenin. Likewise, in a subset of HCC, activating mutations in *CTNNB1* prevent phosphorylation and subsequent degradation of β-catenin, stabilizing it even in the absence of the Wnt ligand. Cytoplasmic β-catenin can then translocate into the nucleus where it activates transcription of target genes that promote cell cycle progression, cell survival, and metabolic changes.

*CTNNB1* mutations occur early during hepatic carcinogenesis and are trunk mutations, found ubiquitously in most cells within the tumor with minimal ITH at the genome level [13]. However, several studies have documented phenotypic/functional ITH in the form of varying levels of Wnt/β-catenin signaling among tumor cells. Rebouissou et al. showed that hepatocellular adenomas (HCAs) and HCCs with similar *CTNNB1* mutational status can display varying levels of β-catenin nuclear localization and target gene expression [62]. Likewise, Friemel et al. showed that in two HCC samples taken from the same liver, tissue sections with identical *CTNNB1* mutations showed heterogeneous nuclear β-catenin staining [68]. HCCs harboring strongly activating *CTNNB1* mutations displayed homogenously strong nuclear β-catenin immunohistochemical (IHC) staining and homogeneously strong and diffuse IHC staining for the β-catenin target protein glutamine synthase (GS), indicating low ITH with respect to Wnt/β-catenin pathway activation. In contrast, HCCs containing weakly activating mutations affecting the S45 residue of CTNNB1 displayed heterogeneous GS staining and absent or rare β-catenin positive nuclei by IHC, indicating considerable phenotypic ITH [68].

Studies in animal models further support a role for Wnt/β-catenin signaling ITH in HCC. Using fluorescent reporter lines and RNAseq analysis, we found that zebrafish HCCs display ITH with respect to β-catenin nuclear and cytoplasmic localization and target gene expression. Although transgenic zebrafish HCC contain the mutated *ctnnb1* transgene in all tumor cells, β-catenin nuclear and/or cytoplasmic staining was only observed in scattered HCC cells [69]. Similarly, only 0.8% to 2.2% of hepatocytes expressed three or more of the Wnt/β-catenin target genes *axin2*, *mtor*, *glula*, *myca*, and *wif1* [70]. These findings support the hypothesis that the IHC heterogeneity observed in human tumors is due to true differences in Wnt/β-catenin signaling rather than to artifacts of the IHC approaches used in patient studies.

In an elegant demonstration of genetic similarity and phenotypic diversity, Brabletz et al. showed that colon cancer cell lines with mutations that inactivate *APC* or stabilize β-catenin have the ability to assume at least two different phenotypes [71]. At low density and soon after seeding, *APC* mutation-containing SW480 cells and *CTNNB1* mutation-containing LS174T cells both assumed a mesenchyme-like phenotype with strong nuclear/weak cytoplasmic β-catenin localization and perinuclear E-cadherin localization. With increasing density, both cell types acquired an epithelial-like phenotype with β-catenin translocating from the nucleus to the cytoplasm and membrane and E-cadherin translocating from the perinuclear region to the membrane and membrane-adjacent cytoplasm. Co-culturing of SW480 or LS174T cells with fibroblast spheroids resulted in SW480 or LS174T cells without nuclear β-catenin clustering around the spheroids. Strong nuclear β-catenin was observed in SW480 or LS174T cells proximal to the fibroblasts and in those cells infiltrating the spheroids, suggesting that nuclear localization of β-catenin may also be an indicator of tumor invasiveness. In accordance with this finding, strong nuclear β-catenin localization was predominantly confined to tumor cells at the invasion front in clinical colorectal carcinoma [71,72]. From these observations, and given the largely conserved signaling mechanisms of the Wnt/β-catenin pathway across different tissue types, it may be reasonable to infer that HCCs that display ITH with respect to subcellular β-catenin localization may contain tumor subpopulations with varying propensities for invasion and metastasis.

Selective activation of the Wnt/β-catenin pathway is critical for metabolic zonation during liver development, with pericentral hepatocytes requiring active β-catenin and periportal hepatocytes expressing the negative regulator *APC*. Mice with hepatocyte-specific loss of *APC* showed upregulation of pericentral and downregulation of periportal genes across the entire hepatic lobule, indicating loss of zonation/metabolic heterogeneity [73]. Additionally, inhibition of the Wnt/β-catenin pathway via ablating Wnt ligand expression in hepatocyte-adjacent endothelial cells resulted in impaired metabolic zonation with loss of pericentral β-catenin target genes (*Gs*, *Axin2*, and *Cyp2e1* cytochrome P450 2E1) and expression of periportal genes (*Arg1*, arginase 1) [74]. These studies position β-catenin as a critical regulator of hepatic metabolism whose expression in the liver is inherently heterogeneous, and they suggest that HCC subpopulations exhibiting heterogeneous β-catenin activity might possess varying metabolic characteristics as well.

The aforementioned studies underscore the functional ITH that may arise even in the case of trunk mutated oncogenes such as *CTNNB1* and suggest that this ITH can be indicative of HCC tumor subpopulations with varying degrees of invasiveness and aberrant metabolism. Given the well-established role of the Wnt/β-catenin pathway in stem cell self-renewal capacity in breast, lung, and intestinal tumors [75,76] and the implication of β-catenin in diminishing immune surveillance in HCC [77,78], it is tempting to speculate that β-catenin ITH might also induce gradients with respect to stemness and immune cell recruitment in HCC. Further studies in animal models could focus on testing this hypothesis and defining how β-catenin-driven ITH in stemness and immune cell recruitment influences tumor behavior and therapeutic response.

### 7.2. TERT ITH

Telomerase reverse transcriptase (TERT) is the catalytic subunit of the telomerase enzyme and regulates its activity [79,80]. In addition to its role in telomere maintenance, TERT has non-canonical functions in stress response, metabolism, and signal transduction that are independent of its telomerase activity [81,82]. Increased *TERT* expression promotes telomerase activation, cell proliferation and immortalization, and tumorigenesis [79]. *TERT* promoter mutations (TPMs) that increase TERT expression are found in several cancers, including HCC [61], melanoma [83], basal and squamous cell carcinoma [84], glioblastoma [85], and bladder [86] and thyroid cancer [87].

Kwa et al. recently investigated the association between TPMs and ITH in HCC, categorizing 97 HCC specimens as TPM-positive or TPM-negative [88]. While all HCCs had large regions of moderate differentiation, TPM-positive HCC contained more extensive well-differentiated and poorly differentiated regions than TPM-negative HCC. Thus, TPM-positive HCCs were more histologically heterogeneous. The importance of this histologic ITH to HCC prognosis and therapeutic response is yet to be explored.

Meningiomas display ITH with respect to *TERT* expression. Abedalthagafi et al. presented a case of a patient with intraventricular meningioma with morphological heterogeneity and found that *TERT* mRNA expression was low in grade I area of the meningioma but identified the same TPM (C228T) in both the grade II and III sections leading to increased *TERT* expression in high-grade areas [89]. Unsurprisingly, given this association with high-grade morphology, TPMs were associated with shorter overall survival in meningioma patients [90]. Although TPMs are not associated with prognosis in HCC, longer telomere length is associated with poor overall survival [91]. It will be useful for future studies to determine if TPMs, *TERT* expression, and TERT functionality are heterogeneous in HCC patients and to define if this *TERT* ITH affects their prognosis.

## 8. Clinical Consequences of ITH for HCC

ITH presents several challenges for HCC diagnosis, prognosis, and therapy. As demonstrated by Xue et al. [15], distinct lesions from a single HCC patient can possess different genetic landscapes, implying that molecular analyses of cells derived from single-region biopsies can misrepresent tumor properties, incorrectly biasing clinical decisions. Even in the case of solitary tumor nodules, single-region biopsies may not capture subclonal mutations that might serve as druggable targets or sources of potential therapeutic resistance. ITH is even more important to investigate in the setting of multifocal HCC as each focus can be composed of subclones, harboring potentially clinically relevant aberrations, as highlighted by Xu et al. [13]. Multiregional biopsies can be technically challenging to perform in solid tumors, and there is a relatively high risk of bleeding in HCC patients who usually have chronic liver disease.

Liquid biopsies are recent additions to efforts to measure ITH, leveraging the availability of circulating tumor cells, DNA, and/or T-cell antigen receptors. Since the release of these molecules into the bloodstream by tumors is not fully characterized or uniform, findings from these measurements may need to be interpreted with caution. However, Huang et al. found that more than 80% of mutations in *TP53*, *TERT*, and *CTNNB1* in circulating tumor DNA were concordant with their status in paired HCC tumor tissue [92]. Future studies will be required to determine if more heterogeneous mutations such as those in *PIK3CA* are well-represented by liquid biopsy.

Following assessment of ITH, the prediction of how the identified ITH will impact clinical outcomes is not straightforward. Measurement of ITH results in a steep increase in the number of variables that need to be considered when building mathematical models to forecast clinical outcome. Recent efforts begin to address this computational challenge. The DARWIN clinical trial aims to evaluate whether targeting a clonal versus subclonal mutation with the anti-PD-L1 drug atezolizumab alters progression-free survival in patients with non-small-cell lung cancer (NCT02183883). Lin et al. [29] used the MATH algorithm to demonstrate that higher methylation ITH scores may indicate worse survival outcomes for HCC patients. Further studies directly investigating the relationship between ITH and patient outcome in HCC are critical to determine how widely the MATH algorithm can be applied and to developing improved models for predicting clinical outcomes based in ITH.

ITH plays a critical role in therapeutic resistance via various potential mechanisms, so careful consideration of ITH is helpful when formulating clinical strategies [5,7] (Figure 2). Tumor cells can develop drug resistance through genetic amplification of the therapeutic target, point mutations that affect the ability of the therapeutic to inhibit the oncogenic pathway, and/or amplification/inhibition of other genes that compensate for the drug-inhibited oncogene [93]. When devising treatment strategies for heterogeneous HCCs, it is important to consider that each cell within a single tumor could respond differentially to therapeutic stress via one or more of these resistance mechanisms. With treatment, pre-existing, undiagnosed, and untargeted ITH can result in the expansion of smaller subclonal population(s) as a result of positive selection, with the destruction of drug-responsive subclone(s) [5]. Alternatively, therapy can induce a subset of cells within the tumor to undergo resistance-conferring epigenetic changes [5]. Clonal cooperation is also thought to influence drug sensitivity [6]. In HCC, long-term treatment with sorafenib promotes epithelial-to-mesenchymal transition via the PI3 kinase/AKT-snail pathway in vitro [94], and tumors resistant to sorafenib upregulate insulin-like and fibroblast growth factor signaling pathways in vivo in animal models [95]. Further studies in animal models defining the degree of ITH in HCC before and after sorafenib administration will help elucidate which resistance mechanisms are most relevant to HCC and help determine how post-sorafenib ITH affects response to second-line therapies.

## 9. Conclusions

Knowledge of ITH corroborates the inherent complexity of HCC biology and therapy response, but it presents many clinical challenges. Nonetheless, it brings several unprecedented opportunities to the fore. The assessment of ITH allows for more informed and adaptable clinical decision making at various stages of tumor progression with respect to choosing therapy, predicting therapeutic efficacy, and revising treatment strategy post resistance. ITH data also provide insight into the functioning of a tumor as an ecosystem and inspires the development of mathematical models to more accurately predict clinical outcome.
